# Human Coronaviruses: Counteracting the Damage by Storm

**DOI:** 10.3390/v13081457

**Published:** 2021-07-27

**Authors:** Dewald Schoeman, Burtram C. Fielding

**Affiliations:** Molecular Biology and Virology Research Laboratory, Department of Medical Biosciences, University of the Western Cape, Cape Town 7535, South Africa; dschoeman@uwc.ac.za

**Keywords:** human coronaviruses, cytokine storm, immunopathology, COVID-19

## Abstract

Over the past 18 years, three highly pathogenic human (h) coronaviruses (CoVs) have caused severe outbreaks, the most recent causative agent, SARS-CoV-2, being the first to cause a pandemic. Although much progress has been made since the COVID-19 pandemic started, much about SARS-CoV-2 and its disease, COVID-19, is still poorly understood. The highly pathogenic hCoVs differ in some respects, but also share some similarities in clinical presentation, the risk factors associated with severe disease, and the characteristic immunopathology associated with the progression to severe disease. This review aims to highlight these overlapping aspects of the highly pathogenic hCoVs—SARS-CoV, MERS-CoV, and SARS-CoV-2—briefly discussing the importance of an appropriately regulated immune response; how the immune response to these highly pathogenic hCoVs might be dysregulated through interferon (IFN) inhibition, antibody-dependent enhancement (ADE), and long non-coding RNA (lncRNA); and how these could link to the ensuing cytokine storm. The treatment approaches to highly pathogenic hCoV infections are discussed and it is suggested that a greater focus be placed on T-cell vaccines that elicit a cell-mediated immune response, using rapamycin as a potential agent to improve vaccine responses in the elderly and obese, and the potential of stapled peptides as antiviral agents.

## 1. Introduction

Coronaviruses (CoVs) are enveloped viruses with single-stranded, positive-sense RNA that belong to the family *Coronaviridae*, under the order *Nidovirales*. With genomes ranging from 27 to 31 kb (kilobases) in size, CoVs have the largest genomes of RNA viruses [1]. Traditionally, CoVs infect various animals, including birds, cats, dogs, and farm animals such as horses, cattle, and swine [2,3]. The last few decades, however, have seen some CoVs traverse the animal–human species barrier, to give rise to human (h) CoV (hCoV) infections.

To date, seven hCoVs have been identified as causative agents of acute respiratory tract infections (ARTIs) [4,5]. Four of these hCoVs, viz. HCoV-229E, HCoV-NL63, HCoV-OC43, and HCoV-HKU1, are less pathogenic and typically cause mild, self-limiting upper respiratory tract infections (URTIs) in immunocompetent persons, which can resolve without any therapeutic intervention. However, in persons with compromised immune systems or chronic comorbidities, such as cancer, transplant recipients, or chronic obstructive pulmonary disease (COPD), these hCoV infections exacerbate pre-existing conditions and lead to more serious lower respiratory tract infections (LRTIs) [6,7,8,9,10]. These less pathogenic hCoVs reportedly do not have animal reservoirs and are well-adapted to humans, circulating seasonally within the global population [7,11,12].

Conversely, the other three hCoVs, viz. severe acute respiratory syndrome (SARS)-CoV, Middle East respiratory syndrome (MERS)-CoV, and SARS-CoV-2, are more pathogenic and have caused severe outbreaks in the last two decades. The SARS-CoV outbreak of 2003 was the first severe outbreak caused by a highly pathogenic hCoV, with 8096 laboratory-confirmed cases and 774 deaths reported worldwide—a case-fatality rate (CFR) of 9.6% [13]. In Saudi-Arabia, 2012, an outbreak caused by the MERS-CoV resulted in 2499 laboratory-confirmed cases with 861 associated deaths, with a CFR of 34.4% as of December 2019 [14]. More recently, in late 2019, the coronavirus disease 2019 (COVID-19) outbreak originated in Wuhan, China and SARS-CoV-2 (formerly 2019-novel CoV) was subsequently identified as the causative agent and the latest highly pathogenic hCoV [15]. As of writing, there have been 119,212,530 confirmed cases of COVID-19 with at least 2,642,612 deaths reported globally [16].

Those infected with these highly pathogenic hCoVs can exhibit an array of clinical manifestations, the majority of whom experience moderate illness for a short time period, while a small, but substantial number of patients experience a more severe form of the disease characterised by acute lung injury (ALI) that often culminates in acute respiratory distress syndrome (ARDS) followed by death [17,18,19,20,21,22,23,24]. This essentially creates the following two groups of patients: those who develop mild disease and recover, and those who develop a more severe disease and succumb to the infection. All three highly pathogenic hCoVs are also known to induce elevated levels of inflammatory cytokines and chemokines [25,26,27,28,29] that have been associated with ALI and ARDS seen in severe cases of the disease [26,28,30,31]. Several other factors have also been shown to influence the severity of disease in infected persons, including age, sex, and comorbidities. Younger persons were less frequently infected and less likely to experience severe disease compared to adults and the elderly; SARS-CoV infections showed a female predominance, whereas MERS-CoV and SARS-CoV-2 reflected a higher incidence of male infections; individuals with comorbidities were more likely to experience severe disease [24]. Furthermore, asymptomatic infections have been reported for SARS-CoV, MERS-CoV, and SARS-CoV-2, which is of great concern since such infections can contribute to the transmission of highly pathogenic hCoVs and pose a challenge to infection control and a threat to public health [32,33,34,35,36].

Interestingly, it seems that the transmissibility differs between each of the highly pathogenic hCoVs. Having infected approximately 2499 persons between 2012 and 2019, the transmission of MERS-CoV between humans seems to be relatively ineffective, compared to SARS-CoV, which infected 8096 people over a span of six months. Conversely, SARS-CoV-2 is the first hCoV to cause a pandemic and has, to date, infected significantly more people than both its predecessors combined in a little more than one year, suggesting that it transmits more effectively between humans than either SARS- or MERS-CoV. This could, in part, be due to the reported greater affinity of the SARS-CoV-2 spike (S) protein for the angiotensin-converting enzyme 2 (ACE2) receptor, in addition to other factors [37]. However, the inverse seems to be true of the respective CFRs, where MERS-CoV exhibits the highest CFR, followed by SARS-CoV, with SARS-CoV-2 having the lowest CFR among the highly pathogenic hCoVs. This would appear as though there might be a possible trade-off between viral transmission and host mortality, but admittedly more research would be required to determine whether there could actually be any possible relationship between virus transmissibility and host mortality.

## 2. Clinical Presentation of Highly Pathogenic hCoV Infections

The clinical presentation, and laboratory and imaging abnormalities among SARS-CoV, MERS-CoV, and SARS-CoV-2, are not disease-specific, but rather resemble most typical acute LRTIs. Diseases SARS, MERS, and COVID-19 demonstrate an almost equally wide clinical spectrum, ranging from asymptomatic, subclinical, and self-limiting disease to severe disease and ARDS, most often seen in older persons and those with chronic comorbidities [20,38,39]. A comparison of the epidemiological, clinical, and laboratory features between SARS, MERS, and COVID-19 can be found in Table 1.

### 2.1. SARS

The mean estimated incubation period for SARS is 4.6 days, with the majority manifesting symptoms within 10 days after infection, and where hospitalisation occurs between 2 and 8 days after illness onset [56]. Generally, SARS clinically presents with persistent fever, rigour or chills, myalgia, dry cough, malaise, headache, and dyspnoea. To a lesser extent, sputum production, sore throat, and coryza have also been reported. The clinical course of SARS is mild in children younger than 12 years old with no mortality, whereas adults typically exhibit a clinical course pattern that can be grouped into three distinct phases [57,58]. During the first week (phase one) the viral load increases by viral replication and is accompanied by the clinical features fever, cough, myalgia, and other systemic symptoms. However, these generally improve after a few days. Despite a subsequent, progressive decrease in viral load, immunopathological damage sets in (phase two), characterised by a recurrence of fever, oxygen desaturation, and radiological progression of pneumonia [39,58,59,60]. Approximately 20% of patients progress to phase three, during which ARDS occurs and often culminates in death [61,62]. There have also been some reports of gastrointestinal symptoms such as abdominal pain, vomiting, and diarrhoea [56,59].

### 2.2. MERS

The incubation period of primary MERS cases is difficult to establish since it is not known what leads to sporadic MERS-CoV infection. However, data from several clusters of human–human transmission estimates incubation to be over five days and up to two weeks [55]. Nevertheless, MERS most commonly manifests with flu-like symptoms such as fever, myalgia, sore throat, non-productive cough, shortness of breath, and dyspnoea, which rapidly progresses to pneumonia [19,63]. Some atypical symptoms have also been reported to include chills, wheezing, palpitations, and mild respiratory illness in the absence of fever, as well as some gastrointestinal symptoms such as abdominal pain, nausea, vomiting, and diarrhoea [20,64,65]. The majority of patients who contract MERS develop severe pneumonia and require ICU admission [20,66]. The more severe MERS cases were those reported in primary index cases, immunocompromised persons, and those with comorbidities, whereas secondary MERS cases (i.e., household contacts or healthcare workers) only exhibited mild respiratory illness or were mostly asymptomatic [67].

### 2.3. COVID-19

The typical incubation period for COVID-19 is between 2 and 14 days, underpinning the rationale behind the 14-day self-quarantine period [68]. Given the effect of the COVID-19 pandemic on the world, the clinical presentation of COVID-19 has become extensive, but the focus here will only be on the general clinical presentation of the disease. Regardless, there is quite a bit of overlap between the clinical manifestations of the highly pathogenic hCoV infections. Patients with COVID-19 often present with symptoms that resemble the flu and typically include fever, cough (some with sputum production and others not), sore throat, dyspnoea, and nasal congestion. Some gastrointestinal symptoms also include nausea, vomiting, and diarrhoea [40,69,70,71]. Ground glass opacity and bilateral patchy shadowing were frequently reported radiographical abnormalities, while other findings included interlobular septal thickening and reticular pattern [69,72]. Interestingly, a small proportion of both severe and non-severe patients did not exhibit any detectable radiological abnormalities [69]. A comprehensive review of the clinical presentation of COVID-19 for various age groups and the different organ systems can be found in [41]. Similar to SARS and MERS, age and comorbidities have been linked to more severe outcomes [38,68]. Furthermore, asymptomatic transmission is of great concern in the driving of the COVID-19 pandemic [73].

## 3. Lung Pathology of Highly Pathogenic hCoV Infections

### 3.1. Viral Tropism and Pulmonary Histopathological Features of SARS

The detection of a viral antigen in airway and alveolar epithelial cells, vascular endothelial cells, and macrophages [74,75], as well as virions in pneumocytes and alveolar macrophages [76], demonstrates clear evidence of SARS-CoV tropism for pulmonary tissue and the ensuing pathology. However, viral particles and viral genome have also been detected in circulating monocytes and lymphocytes, demonstrating that viral tropism extends beyond the lungs and could be linked to the manifestation of extrapulmonary symptoms or complications [74,77,78]. Data on the pathological changes due to SARS-CoV infection is based on autopsies. Gross examination of lungs showed an increased weight, were oedematous, and exhibited extensive consolidation [74,75,79,80,81].

The pulmonary histopathology of patients who succumbed to SARS generally showed lung consolidation and oedema with plural effusions, focal haemorrhages, and mucopurulent material in the tracheobronchial tree. A defining feature of SARS-CoV infections was diffuse alveolar damage (DAD) [74,75]. The following characteristics of acute DAD could be observed during the first 7–10 days of the infection: extensive oedema, hyaline membrane formation, alveolar collapse, alveolar epithelial cell desquamation, and the presence of fibrous tissue in alveolar spaces [79,82,83,84]. In the event of a longer disease duration, features of the fibrous organisation of DAD, and interstitial and airspace fibrosis and the pneumocytic organisation of lung tissues become apparent after 10–14 days [30,76,84,85,86]. In later stages, additional features such as hyaline membrane formation, alveolar haemorrhage, and fibrin exudation in alveolar spaces with septal and alveolar fibrosis could be observed [62,75]. Further histological examination of the SARS-CoV-infected lungs revealed extensive interstitial and alveolar infiltration of neutrophils and macrophages, with macrophages being the predominant cellular infiltrate [74,75]. In SARS cases that have lasted 2–3 weeks, dense septal and alveolar fibrosis was observed [30,76,86], demonstrating that a longer duration of the disease produces a more extensive fibrous organisation of lung tissue [83,84].

### 3.2. Viral Tropism and Pulmonary Histopathological Features of MERS

Pulmonary cells pneumocytes, multinucleated epithelial cells, and bronchial submucosal gland cells that express the MERS-CoV host cell receptor, dipeptidyl peptidase 4 (DPP4) [87], have been identified as targets for MERS-CoV infection [88]. Viral particles have also been observed in pneumocytes, pulmonary macrophages, macrophages that infiltrate skeletal muscles, and renal proximal tubular epithelial cells [89]. Due to cultural reasons in the Arabian Peninsula, human autopsies were rarely performed on MERS patients, thereby limiting the available human data to a few studies [90]. Human autopsies have reported exudative DAD, similar to SARS, accompanied by the formation of hyaline membranes, pulmonary oedema, type 2 pneumocyte hyperplasia, interstitial pneumonia of a predominantly lymphocytic nature, and multinucleate syncytial cells [88,89]. Furthermore, bronchial mucosal and epithelial cell necrosis, sloughing of bronchiolar epithelium, thickened alveolar septa, and alveolar oedema was also evident [88]. Many of the observed features for MERS overlap with the histopathology observed in SARS cases, apart from some that are most likely attributed to the difference in the host cell surface receptor. Animal studies from several different species have provided additional histopathological data [91,92,93,94,95,96,97], and although many species have resembled the features obtained from human autopsies, the outcomes varied between animals [98].

### 3.3. Viral Tropism and Pulmonary Histopathological Features of COVID-19

Similar to SARS-CoV, SARS-CoV-2 binds to the ACE2 receptor [37], and therefore, a similar viral tropism is to be expected [99]. Autopsies characteristically demonstrated DAD much like SARS-CoV and MERS-CoV infections. However, the morphological features may vary depending on the duration of the disease. During the first week after pulmonary injury, the acute/exudative phase, intra-alveolar oedema, and interstitial widening are evident along with diffuse or focal hyaline membranes. Inflammation is generally low during this phase and thrombi may be present [100]. The subsequent, second phase of DAD is known as the organising/proliferative phase, which is characterised by cellular fibroblastic proliferation. While the hyaline membranes disappear as they become integrated into the alveolar septa during this phase, it is also associated with hyperplasia of type II pneumocytes and squamous metaplasia. Following the organising phase, patients can either experience a gradual resolution of DAD or develop interstitial fibrosis. Regardless, most survivors experience some kind of functional impairment of the lungs [100].

## 4. Cytokine and Chemokine Responses of Highly Pathogenic hCoV Infections

### 4.1. Cytokine and Chemokine Response to SARS-CoV Infection

Pro-inflammatory cytokines and chemokines are notably upregulated in SARS patients. Cytokines such as interleukin (IL)-1β, IL-6, IL-12, interferon-gamma (IFN-γ), transforming growth factor beta (TGF-β), and IFN-α and chemokines C–C motif chemokine ligand 2 (CCL2), C-X-C motif chemokine ligand 9 (CXCL9), CXCL10, macrophage inflammatory protein-1 alpha (MIP-1α), and IL-8 have been reported to be substantially elevated in severe SARS patients [25,26,101,102], whereas mild patients were found to have an increased production of T-helper 1 cell (Th1)-related cytokines IL-2, IL-12, IFN-γ, and tumour necrosis factor alpha (TNF-α) [54,67].

It should be noted that these studies have monitored the cytokine and chemokine levels of SARS patients but have not always included a standardised, consistent cytokine panel, making it a challenge to draw absolute conclusions from the cytokine profiles and their relevance in disease progression or resolution. Granted, factors such as constrained resources are a realistic hindrance that can hamper a comprehensive evaluation of the immune response in patients. However, when analysing such data, it would be prudent to take into consideration the phase of the clinical course, disease severity, types of specimens collected, cytokine panels assessed, detection method(s) used, and the previous medication of patients, as these might affect the inferences that can be drawn from such data [54].

### 4.2. Cytokine and Chemokine Response to MERS-CoV Infection

With the wide expression of the MERS-CoV DPP4 host entry receptor, it stands to reason that MERS-CoV can infect a wider range of host cells [103], including activated leukocytes [104]. In fact, MERS-CoV could even infect dendritic cells [105], macrophages [106], and T cells [107], whereas SARS-CoV caused abortive infections in these cells. MERS-CoV is able to infect both dendritic cells and macrophages (naïve and activated), inducing a robust and sustained production of pro-inflammatory cytokines and chemokines, including TNF-α, IL-6, CXCL10, CCL2, CCL3, CCL5, and IL-8 [105,106]. Higher levels of pro-inflammatory cytokines (IL-1β, IL-6, IL-8) and attenuated antiviral cytokines (TNF-α, IP-10, IFN-β) were observed in acute phase MERS patients compared to SARS patients [108]. This could be attributed to the wider distribution of DPP4 receptors in various tissues [109,110,111], compared to ACE2 receptors for SARS-CoV infection [112], creating a bigger probability of cytokine production in MERS than in SARS. Interestingly, MERS-CoV was able to infect T cells more efficiently than SARS-CoV, specifically the CD4^+^ Th-cells more so than the CD8^+^ cytotoxic T lymphocytes (CTLs). While SARS-CoV was unable to infect T cells, the MERS-CoV infection of T cells was efficient, but abortive, and induced apoptosis via the extrinsic and intrinsic pathways. This is thought to be a mechanism of T-cell evasion by MERS-CoV that possibly contributes to the systemic dissemination and immunopathology caused by the virus [107] and a way in which host cells could serve as reservoirs to shield the virus from the immune system [105].

### 4.3. Cytokine and Chemokine Response to SARS-CoV-2 Infection

Similar to SARS-CoV and MERS-CoV infections, various inflammatory mediators, cytokines and chemokines were elevated in COVID-19 patients. Patients with severe COVID-19 have been reported to exhibit elevated levels of IL-1β, IL-1Ra, IL-2, IL-7, IL-8, IL-9, IL-12, IL-13, IL-17, IFN-γ, granulocyte colony-stimulating factor (GCSF), macrophage colony-stimulating factor (MCSF), hepatocyte growth factor (HGF), MIP-1α, IP-10, MCP-1, MCP-3, and TNF-α [28,113,114], compared to healthy controls. The patients who were admitted to the ICU were also found to have higher plasma levels of IL-2, IL-7, IL-10, GCSF, IP-10, MCP-1, MIP-1α, and TNF-α than non-ICU patients [113]. Interestingly, while many of these cytokines and chemokines were also elevated in SARS and MERS patients, several studies have reported an increase in Th2-related anti-inflammatory cytokines IL-4 and IL-10 [115] in COVID-19 patients [113,116,117,118,119,120], ascribing a seemingly unique cytokine profile to COVID-19. Even more interesting is that IL-10 levels were increased in severe COVID-19 patients, compared to the mild patients [113,116,117,118,119,120]. The induction of overlapping cytokines in SARS-CoV, MERS-CoV, and SARS-CoV-2 infections suggests a possible common immunopathogenic pathway induced by these highly pathogenic hCoVs. Some overlapping features can be attributed to the genetic similarity between these hCoVs [121], whereas distinct properties for each hCoV could be attributed to the difference in host cell receptor and viral tropism [122,123,124,125] or the number and function of accessory proteins [126]. It would be interesting to see which patients had increased IL-10 levels in order to establish a possible underlying mechanism or pathway. Were the increased levels the result of comorbidities, the virus itself, or the patient’s immunological profile, viz. B- and T-cell and major histocompatibility complex repertoire? Such analyses would help to elucidate the underlying pathophysiology and perhaps even the cellular source that is responsible for the increase in IL-10 levels.

The increase in IL-10 could be regarded as a possible negative feedback mechanism to mitigate the effects of inflammatory cytokines such as IL-6 [127,128,129,130]. In fact, the absence of IL-10 in the presence of CXCL10, IL-2, and IL-6 in SARS infections was thought to contribute to its immunopathology [101]. However, this same ability of IL-10 to suppress inflammation and inhibit the function and proliferation of immune cells might also be detrimental to the host since it could also hinder viral clearance [130]. In COVID-19, IL-10 levels were sustained for the duration of the disease, rising as early as the first week in patients who went on to develop severe disease [131], whereas IL-10 levels only increased in convalescent SARS patients. The latter response fits well into the expected progression and resolution of the SARS infection, allowing IL-10 to resolve the inflammatory response and ensure homeostasis is maintained following the eradication of the SARS-CoV virus. Conversely, in COVID-19, the early induction of and sustained IL-10 levels might delay the onset of symptoms, possibly protecting the host against any potential initial immune-mediated complications. While this response might be beneficial at the onset of symptoms, the sustained elevation of IL-10 for the duration of the disease is likely to impede the development of an effective adaptive immune response capable of completely eradicating the SARS-CoV-2 virus. This would suggest that the early induction of IL-10 in COVID-19 appears to be able to modify the course of the infection during the early stages of the disease, and certainly has implications for the therapeutic use of IL-10 in severe COVID-19 patients. Since this phenomenon appears to be unique to COVID-19, as opposed to other highly pathogenic hCoV infections, it stands to reason that SARS-CoV-2 could induce an early and sustained induction of IL-10 by some mechanism or pathway that neither SARS-CoV nor MERS-CoV is able to. It would certainly prove interesting and understanding how SARS-CoV-2 is able to do this would be beneficial to patient management and could have potential therapeutic applications for COVID-19.

## 5. A Dysregulated Immune Response in Highly Pathogenic hCoV Infections

During an infection, the various cells of the immune system coordinate an appropriate response by secreting cytokines—intercellular signalling molecules that function to induce the proliferation and differentiation of the specific immune cells required to eradicate the infection. Cytokines also serve to regulate the immune response, with some stimulating or escalating the response, while others inhibit and deescalate the response. Ideally, the immune system should recognise a pathogen, respond proportionally to the pathogenic burden, and return to homeostasis once the pathogen has been successfully eradiated. This would require a balanced response in which sufficient cytokines are produced to eliminate the invading pathogen, while simultaneously avoiding a hyperinflammatory response in which excessive cytokines would cause clinically significant collateral damage to the host, ensuring a protective role, rather than a pathogenic one [132]. Cytokine secretion, although most commonly associated with infections, have also been reported in other diseases, including COPDs, such as asthma, various autoimmune diseases, some neurological disorders, and some therapies [133,134]. In such cases, the necessity of proper immune regulation becomes clear, without which the host suffers pathology mediated by the same system purposed to protect it. Essential to immune regulation is negative feedback systems, such as regulatory cell types, anti-inflammatory cytokines (e.g., IL-10 and TGF-β), and decoy receptors for pro-inflammatory cytokines (e.g., IL-1Ra), to antagonise inflammatory cell populations and prevent a hyperactive immune response [132].

Several viruses, such SARS-CoV, MERS-CoV, avian influenza, and the Ebola virus, are known to hyperstimulate the immune system and induce a cytokine storm [135], in which the elevated levels of cytokines and chemokines have been linked to disease severity and clinical progression [25,114,136]. Both SARS-CoV and MERS-CoV can replicate to high viral titres early on in infection [58,137,138,139,140], which, in turn, can induce the production of inflammatory cytokines and chemokines from infected cells [139,141,142], leading to the infiltration of inflammatory cells such as neutrophils and macrophages [137,143]. At the same time, both SARS-CoV and MERS-CoV encode several viral proteins that can subvert both the antiviral IFN response and the subsequent IFN-stimulated genes (ISGs) [144,145,146,147,148,149]. This IFN response is crucial to initiating the appropriate immune response to these infections as it is responsible for orchestrating the proper progression from the innate to the adaptive immune system to ensure successful viral clearance [150,151]. The initial IFN inhibition caused by these viral proteins produces a delayed IFN response, which, in turn, produces a dysregulated antiviral immune response. In fact, the excessive inflammatory response and concomitant lung immunopathology and lethal pneumonia in SARS-CoV infection have been attributed to this delayed IFN-I response [137]. Conversely, an early IFN-I response, and even the absence thereof, resulted in a mild clinical disease for SARS in the same mouse model.

Similarly, in MERS-CoV infections, an early IFN-I response served a protective function, whereas a delayed IFN-I response failed to inhibit viral replication effectively and resulted in the recruitment of inflammatory immune cells, which augmented the pro-inflammatory response and culminated in fatal pneumonia [152]. Interestingly, additional features that likely contribute to the severity of MERS-CoV infections, and sets it apart from SARS-CoV infection, is its (1) ability to infect a wider range of host cells and (2) the induction of T-cell apoptosis. Not only could the productive infection of immune cells such as dendritic cells and macrophages shield MERS-CoV from recognition by the immune system, but it allowed these cells to serve as viral reservoirs, permitting the dissemination of MERS-CoV to other organ systems [105]. Furthermore, the infection of T-cells with MERS-CoV was shown to induce T-cell apoptosis through the extrinsic and intrinsic apoptotic pathways [107], enabling the virus to evade another critical antiviral aspect of the immune system as the T-cell response serves a protective role by clearing viral-infected cells [153,154,155]. By inducing T-cell apoptosis, MERS-CoV also indirectly exacerbates the already dysregulated immune response since T-cells also serve a regulatory function in dampening the overreactive immune system [156,157]. Collectively, despite also being able to inhibit the much-needed antiviral IFN response, MERS-CoV clearly possesses additional immune evasion strategies, supporting the severity and higher mortality rate observed in MERS patients compared to SARS.

The dysregulated immune response during COVID-19 has received much attention, and rightfully so, since the hyperinflammatory response associated with it has been linked to disease severity and increased mortality [40,113,114,116]. In COVID-19, elevated cytokine levels and progression of the disease followed a viral load decline, highlighting the dysregulation in the immune response to COVID-19 [158]. Similar to its predecessors, IFN-I signalling is impaired in severe SARS-CoV-2 infections [29,159,160], an immunomodulatory strategy ascribed to the group of β-coronaviruses [159]. However, given the novelty of the virus, not much empirical evidence exists yet to suggest the exact mechanisms behind the immune dysregulation of COVID-19. Proposed mechanisms have mainly been made from inferences based on the genetic similarity between SARS-CoV-2, SARS-CoV, and MERS-CoV [121]. During the incubation period, SARS-CoV-2 has been shown to replicate inside host cells triggering little to no IFN-I response [161]. This could be achieved by strategies akin to CoVs, such as viral RNA synthesis in double-membraned vesicles to conceal viral RNA [162,163] and 5′ capping of viral RNA to mimic host mRNA [164,165,166] to avoid detection by host pattern recognition receptors (PRRs) that would induce an immune response. While the mechanism of inducing T-cell apoptosis is evident in MERS-CoV infections, research on the mechanism behind lymphopenia in SARS-CoV-2 is sparse. Lymphopenia is a hallmark of SARS-CoV-2 infections, with a notable depletion of T-cells, which is associated with severe COVID-19 [167]. However, only a recent preprint has provided evidence that could allude to the possible mechanism behind this T-cell lymphopenia [168]. The study reported elevated levels of TNF-α—which can mediate apoptosis—in their patient cohort and observed significant increases in the percentage of apoptotic cells in the main T-cell subsets. Therefore, an induction of T-cell apoptosis could, similar to MERS-CoV infections, be a mechanism by which SARS-CoV-2 causes a dysregulated immune response, mediated by the elevated plasma levels of TNF-α often seen in severe COVID-19 cases.

Another possible pathway that could cause aberrant cytokine release and potentially contribute to the cytokine storm is antibody-dependent enhancement (ADE). This phenomenon is not uncommon in viral infections, where antibodies capable of binding to these viruses have been reported to facilitate the pathogenesis or severity of the disease caused by the human immunodeficiency virus (HIV) [169,170], Ebola [171,172], influenza [173], and flaviviruses [174]. Recently, antibodies produced against the less pathogenic hCoVs—present in pre-COVID-19 pandemic plasma—were reported to be capable of binding to the SARS-CoV-2 proteins [175,176], suggesting that antibodies from the less pathogenic, seasonal hCoVs might facilitate ADE. In dengue virus infection, ADE has been shown to increase the presentation of viral antigens on the surface of infected cells that, consequently, triggered the release of cytokines such as IFN-γ, TNF-α, IL-1β, IL-2, IL-6, IL-8, and IL-12 [177,178]. Granted, the occurrence of ADE has only largely been limited to in vitro demonstrations and little to no evidence exists to suggest whether SARS-CoV-2 can also induce cytokine and chemokine release through ADE. However, since ADE has been reported in the other highly pathogenic hCoV infections [179,180,181,182,183], it might be worth investigating whether ADE could increase antigen presentation on ADE-permissive cells and, if so, whether this too could cause an aberrant cytokine release that could contribute to the cytokine storm in COVID-19, especially when ADE in COVID-19 could be mediated by the lower affinity antibodies produced against the seasonal hCoVs. Interestingly, the ability of SARS-CoV-2 to, similar to SARS-CoV, infect macrophages and dendritic cells in an abortive manner, could also function to contribute to the cytokine storm. Recently, Zheng et al. [184] reported the ability of SARS-CoV-2 to infect human monocyte-derived macrophages and monocyte-derived dendritic cells. Admittedly, the infection was abortive—as evident by inefficient replication inside these cells—but the infection induced the production of several cytokines, viz. IFN-α, IFN-β, TNF-α, IL-1β, IL-6, IL-10, and the chemokine CXCL10. Additionally, since macrophages with an inflammatory phenotype have been reported to infiltrate the lungs of patients with severe COVID-19 [185,186], it stands to reason that the ability of SARS-CoV-2 to infect alveolar macrophages could contribute to the dysregulated immune response and, ultimately, the cytokine storm seen in severe COVID-19.

It has also become evident that noncoding RNAs play a crucial role in the progression of inflammatory diseases [187,188,189] and might, therefore, be relevant to the cytokine storm in severe COVID-19. While noncoding RNAs have been implicated in a variety of diseases and disorders [190,191,192], long noncoding RNAs (lncRNAs)—RNA transcripts longer than 200 nucleotides—have been strongly implicated in diseases with which the NLRP3 inflammasome and IL-6 are associated [193,194,195] as well as the COVID-19 cytokine storm [196,197]. One study identified several lncRNAs that could target some of the cytokines overexpressed in the COVID-19 cytokine storm [198]. In particular, the authors found that the lncRNA non-coding RNA activated by DNA damage (NORAD) could target cytokines IL-6, IL-10, TNF-α, and chemokine CXCL10—mediators often associated with the cytokine storm. Thus, based on their link to these cytokines, the exact role of NORAD and the other lncRNAs from the study warrants further analysis through in vitro and in vivo studies to determine whether they could potentially serve as biomarkers, prognostic markers, or even drug targets of the COVID-19 cytokine storm. Another study identified four lncRNAs after analysing the transcriptome data from SARS-CoV-2-infected human lung epithelial cell lines and the bronchoalveolar lavage fluid (BALF) of COVID-19 patients and found that these lncRNAs were differentially expressed and strongly correlated with genes involved in cytokine signalling [199]. These cytokine signalling genes are also related to the cytokine storm. The increased expression of the two lncRNAs—wound and keratinocyte migration associated lncRNA 2 (WAKMAR2) and eosinophil granule ontogeny transcript (EGOT)—were suggested to possibly favour SARS-CoV-2 replication in infected cells, whereas it is not known what the roles of the other two lncRNAs—EPB41L4A-AS1 and ENSG00000271646—in anti-viral responses and cytokine signalling are. Interestingly, the authors also found that the dysregulated immune responses in SARS, MERS, and COVID-19 were mediated by different molecular mechanisms, which can also affect the treatment approach for each disease. While most studies have focussed on the role of specific cytokines and other inflammatory mediators, the regulation of the genes involved in the dysregulated immune response has received little attention. Nevertheless, from these studies, it is clear that the cytokine storm encompasses more than just inflammatory mediators at the protein level, even including components involved in the epigenetic and post-transcriptional regulation of immune response genes.

The importance of understanding, and subsequently, mitigating the immunopathology of the dysregulated immune response in severe COVID-19 is underpinned by the consequences of the dysregulated response. Studies have reported that the hyperinflammation and cytokine storm, as a result of the dysregulated immune response, seen in COVID-19 have various consequences, including ALI, ARDS, respiratory failure, and systemic dissemination that could culminate in multiorgan failure [23,129,200,201].

## 6. Treatments of Highly Pathogenic hCoV Infections

### 6.1. SARS-CoV and MERS-CoV: A Historical Perspective

It has been nearly 20 years since the first highly pathogenic hCoV outbreak, SARS, and yet no licenced, commercially available treatment for hCoV infections exists. While several treatments have been researched, none have proven to be successful and effective enough to progress to a large-scale, widely approved treatment. For SARS-CoV infections, treatment attempts have included ribavirin, lopinavir in combination with ritonavir, type I IFNs (IFN-α and IFN-β), convalescent plasma or intravenous immunoglobulin (IVIG), and corticosteroids, but these have largely proven ineffective or, in some cases, even harmful [202,203]. Although treatment attempts for MERS-CoV infections have included some different approaches, such as several antiviral agents, IFNs, fusion inhibitors, cyclophilin inhibitors, and monoclonal antibodies (mAbs), they did not demonstrate a significant benefit in treating MERS in a consistent, controlled manner [203,204]. Consequently, supportive care has become the mainstay treatment for most pathogenic hCoV infections, relying on sufficient rest, proper hydration, and the use of analgesics to provide organ support and manage complications [205]. Several SARS-CoV and MERS-CoV vaccines have also been investigated, some of which show promise in conferring protection against post-vaccine infection challenges [206,207,208,209,210,211]. While vaccines provide essential protection in the absence of effective therapies, the combination of vaccine-induced immunity and successful antivirals should prove even more effective.

### 6.2. SARS-CoV-2

The lack of a licenced, commercially available anti-coronaviral agent has prompted ongoing research for a COVID-19 drug(s) and, as such, several potential therapies have been proposed for COVID-19 [45]. However, several candidates are similar in approach to those that were used for SARS and MERS treatments: broad-spectrum antivirals, fusion-peptide inhibitors, convalescent plasma or IVIG, and corticosteroids [212]. Granted, some of these therapies have proven quite useful given the remarkably rapid spread of COVID-19, and several new compounds have also been investigated for their therapeutic potential compared to those during the SARS and MERS outbreaks. However, the overall approach appears to have stagnated somewhat and while the SARS and MERS treatments might have shown some initial promise, they either never progressed to or passed clinical trials or human-to-human transmission of the virus was disrupted by non-pharmacological methods, and the outbreak, consequently, ended. We did not seem to learn much from the previous hCoV outbreaks and were ill-prepared for the COVID-19 pandemic. While previous treatment approaches have shown promise and were of some value, different approaches or targets should be considered to avoid obtaining similar or equally ineffective results.

### 6.3. Other Prospective Treatments

#### 6.3.1. T-Cell Based Vaccines: The Importance of Cell-Mediated Immunity

Many vaccines stimulate B lymphocytes to produce effective, specific antibodies against the invading pathogen with the aid of Th2 cells. Additionally, while antibodies do play particularly important roles in immunity and fighting infections, they are not always the most effective response to every type of infection [213]. Particularly in viral infections, cell-mediated immunity (CMI) is essential in mediating immunity and successful viral clearance. Antiviral IFNs and Th1 cytokines work synergistically to promote macrophage activity and stimulates CTL activation and proliferation for a specific and effective clearance of the viral pathogen [26]. Thus, T-cell based vaccines—those that induce a potent CD4 and CD8 CMI-mediated antiviral response through the activation and proliferation of CTLs—should be at the forefront of COVID-19 vaccine and/or drug development.

The CoV spike (S) protein has become a popular target for the development of protein subunit vaccines that typically induce robust antibody production with little to no CMI response involving CTLs [214,215,216,217,218,219]. Indeed, such vaccines are valuable in eliciting a protective antibody-mediated immune response and antibodies do have an important role to play in viral infections [220,221,222]. However, CMI is much better equipped at eradicating viral infections and is not known to facilitate viral entry into host cells by which the viral infection is enhanced, as seen in ADE [180,223,224]. In fact, T-cell vaccines mount a more effective antiviral response than antibodies alone by skewing the immune response in favour of producing Th1-based cytokines (IL-2, IL-12, IFN-γ, TNF-α), which, in turn, promote CTL activation and proliferation [225,226,227]. The importance of a CMI response to viral infections, especially CTLs, is exemplified by several studies on viral infections, including SARS-CoV infection [228,229,230,231,232].

To date, some SARS-CoV-2 vaccines have been shown to induce a CMI response. Inovio has developed a SARS-CoV-2 DNA vaccine (INO-4800) that induced both neutralising antibodies and a Th1-skewed response in different animal models [225,233]. This two-dose vaccine has also shown both humoral and T cell responses in 94% of participants in two phase I clinical trials, showing only adverse effects of grade one or below [234]. Similarly, the Pfizer–BioNTech COVID-19 vaccines, BNT162b1 and BNT162b2, were able to induce antibody responses as well as the production of Th1 cytokines in most participants [226,235,236]. The Gam-COVID-Vac (Sputnik V COVID-19) vaccine, consisting of a priming shot with a recombinant adenovirus type 26 vector (rAd26) and a subsequent booster shot of recombinant adenovirus type 5 vector (rAd5), both of which encode the SARS-CoV-2 spike glycoprotein, also induced a CMI response evident by CTL proliferation and IFN-γ production [237,238]. Interestingly, the participants who received the Pfizer–BioNTech (BNT162b2) or the Moderna (mRNA-1273) mRNA vaccine, also showed a three-fold increase in Th1 cell response (CD4^+^ IFN-γ^+^ TNF-α^+^) in response to stimulation with a peptide of the S protein from the less pathogenic HCoV-NL63 [227]. This would suggest that T-cell based vaccines, or at least those similar to BNT162b2 and mRNA-1273, might provide some form of cross-reactivity and may protect against related pathogens. Furthermore, one study reported T-cell memory against SARS-CoV persisting up to 11 years after the initial infection, further corroborating the value of employing T-cell based vaccines to induce a CMI response [239]. Clearly, CMI is a valuable component of the adaptive immune response against viral infections, ensuring successful viral clearance and long-lasting immunological memory. It is, therefore, of paramount importance to conduct further research into the development of vaccines that can harness the full extent of the adaptive immune system instead of solely relying on antibody-mediated protection.

Since the emergence of different SARS-CoV-2 variants, a great emphasis has been placed on the ability of the current market vaccines—of which the Moderna and Pfizer vaccines have received the most attention—to provide sufficient protection against these variants [240]. Although recent studies conducted with different vaccines have reported a moderate decrease in the ability of vaccine-induced antibodies to neutralise some of the variants, the resulting antibodies were still capable of neutralising the variants, retaining a measure of protection [241,242,243,244,245]. Some studies have also reported that when individuals who have previously been infected with SARS-CoV-2 (seropositive) received even a single dose of a vaccine, their antibody levels were similar or higher to those of previously uninfected (seronegative) individuals who received two vaccine doses [246,247,248,249,250]. These studies also demonstrated that a second dose of the vaccine did not significantly increase the antibody titre of seropositive persons. However, this also raised the question, if a single vaccine dose increased the antibody titre of previously infected persons to levels comparable to that of uninfected persons receiving two doses, does it still provide sufficient protection?

It is also concerning that several of the most recent studies appear to solely rely on the antibody response to vaccines as a measure of vaccine efficacy [240,241,242,243,244,246,247,251,252,253,254,255,256,257,258], despite antibodies not being the only component involved in antiviral immunity. Antibodies are indeed a simple, rapid, and reasonably reliable method of determining vaccine efficacy, but several other recent studies definitively highlight the importance of vaccine-induced CMI, especially with the emergence of SARS-CoV-2 variants. It was recently demonstrated that although variants could escape antibody-mediated (humoral) immunity to an extent, vaccine-induced T-cell (cellular) immunity remains largely unaffected by the mutations of variants [259,260,261,262,263,264]. The data from these studies demonstrate that T-cell immunity appears to tolerate the mutations in the S protein of variants more effectively than humoral immunity. This underpins the importance of measuring several aspects of the immune response to the SARS-CoV-2 vaccines, both humoral and cellular. Limiting the response elicited by SARS-CoV-2 vaccines to only one aspect of the immune system—viz. vaccine-induced antibody titres—inaccurately portrays the efficacy of the vaccine and can be misleading to a point where the vaccine appears to be ineffective when, in reality, only the measured response is less effective. Investigating the effects of all the variant mutations on both humoral and cellular immunity would greatly contribute to the development of vaccines that effectively harness the full extent of the immune system rather than relying on a single aspect for protection. Furthermore, if cellular immunity largely remains unaffected by the mutations of recent and possible emerging variants, such data will be of great benefit to dispelling the fear and concerns from the public when new variants emerge as well as vaccine hesitancy.

#### 6.3.2. Rapamycin: Improving Vaccine Responses and Modulating the Cytokine Storm

Although CMI is an essential component to mediating protection in viral infections, antibody-mediated immunity should by no means be neglected. After all, neutralising antibodies block viral entry into host cells [265,266], whereas CMI functions to kill the host cells already infected with viruses [267,268]. Leptin has been reported as a possible mechanism in which the production of protective antibodies in response to vaccination is reduced in obese persons and the elderly [269,270]. Recently, Frasca et al. [271] showed that elevated leptin levels, such as those seen in obese persons and the elderly, cause a decreased expression of both activation-induced cytidine deaminase (AID), the enzyme required for antibody class switch switching, somatic hypermutation, and IgG production, as well as its transcriptional regulator, E47. Furthermore, treating the B lymphocytes from young, lean individuals with leptin also reduced the production of influenza vaccine-specific IgG to levels similar to that of young, obese persons or elderly persons. However, the leptin-induced functionally impaired B lymphocytes could be rescued using rapamycin (RAPA) treatment. The treatment of leptin-induced defective B lymphocytes with RAPA significantly increased the mRNA expression of AID and the production of influenza vaccine-specific IgG. Therefore, since obese persons and the elderly are among the vulnerable populations in the current COVID-19 pandemic, and they are more likely to exhibit impaired antibody responses to vaccines [272,273,274,275], RAPA treatment could possibly be used to enhance the antibody response of these individuals to the COVID-19 vaccine(s).

Furthermore, RAPA (clinically known as Sirolimus or Rapamune) inhibits the mammalian target of rapamycin (mTOR) and has been shown to induce regulatory T cells (Tregs), promote Treg-cell expansion, and suppress effector T cell activation [276,277,278]. The induction and expansion of Tregs is especially important in the regulation of the immune response since they can serve as sources of the anti-inflammatory cytokines IL-10 and TGF-β [279,280,281,282]. In doing so, RAPA could also potentially modulate the cytokine storm by improving the Treg-mediated anti-inflammatory response and dampening the innate and T cell effector-mediated pro-inflammatory response, thereby re-establishing the balance of cytokines to lessen the immunopathology caused by the cytokine storm [283]. Naturally, the extent of Treg cell activation should be carefully considered in order to only trigger a modulatory response to the extent that the cytokine storm-mediated damage is mitigated, while simultaneously being cautious to avoid the induction of a prolonged IL-10 response that might ultimately hinder viral clearance.

#### 6.3.3. Stapled Peptides

Viruses depend on the signalling pathways of their host cells to replicate and propagate [284,285,286], offering several unique targets to disrupt the viral replication cycle and stop the infection. Mainstream antivirals have relied on small-molecule drugs/inhibitors (SMIs) that interfere with crucial virus–host protein–protein interactions (PPIs) that drive the viral replication cycle [287,288]. Indeed, the small size of SMIs make transport across the plasma membrane efficient, but SMIs lack specificity and selectivity for their targets, often causing unwanted side-effects [289]. They also often bind to areas on target proteins that are not involved in PPIs, such as deep grooves or hydrophobic pockets, rendering them less effective at disrupting the desired virus–host PPI [290,291,292]. Conversely, larger, protein-based therapies, viz. growth factors and engineered antibodies, are more selective and potent since they form more interactions and stronger ones with their target protein. Although this decreases the likelihood of side effects, their size severely restricts their passage across the plasma membrane and limits their reach for intracellular targets [289]. Stapled peptides offer overlapping advantages from both SMIs and larger, protein-based therapies, and has already been applied to a variety of human diseases [293,294,295,296,297].

Stapled peptides can be used in several ways [298] but can primarily be designed to be complementary to endogenous ligands, binding to their targets, and inhibiting certain host responses [299]. By binding to target host cell proteins, stapled peptides can potentially be used to activate antiviral cellular pathways or inhibit virus–host PPIs that are crucial to viral infections. Stapled peptides may even be synthesised to serve as “decoys” for viral proteins, allowing viral proteins to bind to other viral or host cell protein decoys without propagating viral replication. The application of stapled peptides in viral infections has largely been limited to HIV-1 [296,300], respiratory syncytial virus (RSV) [301], hepatitis B and C viruses (HBV, HCV) [302,303], and the Ebola and Marburg viruses [304]. At the time of writing, only two papers have investigated the possibility of stapled peptides to inhibit the binding of SARS-CoV-2 to ACE2 receptors [305,306]. Granted, one of these stapled peptides was reported to possess no antiviral activity against SARS-CoV-2 and additional measures were suggested to improve the binding to the target SARS-CoV-2 S-protein RBD and block viral entry [307]. Indeed, stapled peptides are by no means a panacea; in the same way that some drugs work while others fail, some stapled peptides will be effective or only slightly effective [295,308], while others might not be effective at all [307]. Nonetheless, stapled peptides do still offer several advantages. Their design against defined motifs or domains allows them to exhibit greater specificity for their targets; they can target difficult-to-treat—considered “undruggable”—diseases; can be repurposed to enhance membrane permeability, potency, and half-life; and have a short market time [289,298,308,309,310]. These properties, along with their flexibility in synthesis and modification, would make them very valuable as antiviral agents [299,311]. Additionally, similar to SMIs, the repurposing of existing peptides is still a possibility [301].

## 7. Conclusions

The world was ill-prepared for the SARS-CoV-2 outbreak. Although 18 years have elapsed since the first highly pathogenic hCoV outbreak, no licenced, commercially available vaccine or anti-coronaviral treatment has reached fruition, leaving the world to rely on non-pharmacological interventions to mitigate the damage caused by the virus. Yet, a vast amount of research and progress has been accomplished in the short span since the start of the COVID-19 pandemic. Many overlaps were found between COVID-19 and the previous SARS and MERS outbreaks, while some interesting differences have also come to light. One clear lesson is that many of the conventional treatment approaches to the previous outbreaks have largely proven unsuccessful and appear to have a largely similar outcome in treating COVID-19. Another lesson is that the cytokine storm, a driver of disease severity, is far more complex than simply depleting a single cytokine or administering a recombinant one in an attempt to readjust the immune response to achieve homeostasis again. Less conventional treatment approaches should also be given attention and might not only advance therapeutics for COVID-19 but be extended to other infectious diseases as well. Three outbreaks of highly pathogenic hCoVs in nearly 20 years, each more intense than the previous, either in mortality or transmission. If anything, it would be ill-advised not to expect another hCoV outbreak to occur, which is why comprehensive research into the current highly pathogenic hCoVs would be invaluable in preparing for the next possible highly pathogenic hCoV outbreak.

## Figures and Tables

**Table 1:** A comparison of the epidemiological, clinical, and laboratory features between SARS, MERS, and COVID-19 [38,40,41,42,43,44,45,46,47,48,49,50,51,52,53,54]. Adapted from [55].

	SARS	MERS	COVID-19
Date of first case report (place)	November 2002 (China)	April 2012 (Jordan) June 2012 (first KSA case)	December 2019 (Wuhan, China)
Incubation period	Mean: 4.6 days (95% CI: 3.8–5.8)	Mean: 5.2 days (95% CI: 1.7–14.7)	Mean: 5.6–6.7 days (95% CI: 5.2–7.4)
Age (years)	Range: 2–14 days	Range: 2–13 days	Range: 1–14 days
Range, Median	Range: 1–91 Mean: 39.9	Range: 1–94 Median: 50	Range: 16–89 Median: 50
**Mortality**			
Overall case fatality rate (CFR)	9.6%	41.8%	3.8%
CFR in patients with comorbidities	46%	60%	11%
Gender (M, F)	M: 43%, F: 57%	M: 64.5%, F: 35.5%	M: 50.7–56%, F: 44–49.3%
**Presenting Symptoms**			
Fever (>38 °C)	99–100%	98–100%	20–98%
Chills/Rigors	15–73%	87%	11.4–18%
Cough	62–100%	83–100%	28.6–79%
Dry	29–75%	56%	67.7%
Productive	4–29%	44%	23–33.4%
Haemoptysis	0–1%	17%	0.9%
Headache	20–56%	11%	8–15%
Myalgia	45–61%	32%	11–44%
Malaise	31–45%	38%	23.6–34.2%
Shortness of breath	40–42%	72%	4–55%
Nausea	20–35%	21%	2.2–4.5%
Vomiting	20–35%	21%	2.2–4.5%
Diarrhoea	20–25%	26%	5–28.6%
Sore throat	13–25%	14%	11–15%
Rhinorrhoea	2–24%	6%	5.6%
Olfactory/Gustatory dysfunction	N.R. ^1^	N.R. ^1^	64–80%
**Disease Progression**			
Time from onset to ventilatory support	Mean: 11 days	Mean: 7 days	Median: 5 days (IQR: 1–10 days)
Time from onset to death	23.7 days	Median: 11.5 days	Mean: 15.93 days
Comorbidities	10–30%	76%	24–48%
Diabetes	24%	10%	2.7–58%
Chronic renal disease	2–6%	13%	3–13%
Chronic heart disease	10%	7.5%	16.2%
Malignancy	3%	2%	6–8%
Hypertension	19%	34%	4.5–63%
Obesity	N/A	17%	46–47.6%
Smoking	17%	23%	23%
Viral hepatitis	27%	Not known	≤0.1–12.2%
**Imaging and Laboratory Results**			
CXR abnormalities	94–100%	100%	0–63%
Leukopenia (<4.0 × 109/L)	25–35%	14%	31–33.7%
Lymphopenia (<1.5 × 109/L)	68–85%	32%	42–83.2%
Thrombocytopenia (<140 × 109/L)	40–45%	36%	36.2%
Elevated LDH	50–71%	48%	41–75%
Elevated ALT	20–30%	11%	21.3–28%
Elevated AST	20–30%	14%	22.2–35%
Ventilatory support required	14–20%	80%	8–91%
Risk factors associated with poor outcome (severe disease or death)	Advanced age, male gender, high initial or peak LDH, high neutrophil count on presentation, diabetes mellitus or other comorbid conditions, low CD4 and CD8 lymphocyte counts at presentation.	Any immunocompromised state, comorbid illness, concomitant infections, low albumin, age ≥ 65 years.	Advanced age, male gender, comorbidities, immunocompromised state.

^1^ N.R. = not reported.
